# Exploring the role of CHI3L1 in “pre-metastatic” lungs of mammary tumor-bearing mice

**DOI:** 10.3389/fphys.2013.00392

**Published:** 2013-12-25

**Authors:** Stephania Libreros, Ramon Garcia-Areas, Patricia Keating, Roberto Carrio, Vijaya L. Iragavarapu-Charyulu

**Affiliations:** ^1^Department of Biomedical Sciences, College of Medicine, Florida Atlantic UniversityBoca Raton, FL, USA; ^2^Department of Biological Sciences, Florida Atlantic UniversityBoca Raton, FL, USA; ^3^Tumor Immunology Group, Department of Microbiology and Immunology, University of Miami Miller School of MedicineMiami, FL, USA

**Keywords:** CHI3L1, tumor growth, angiogenesis, macrophages, chitin microparticles, pulmonary environment

## Abstract

Elevated levels of chitinase-3-like-1 (CHI3L1) are associated with poor prognosis, shorter recurrence-free intervals and low survival in breast cancer patients. Breast cancer often metastasizes to the lung. We hypothesized that molecules expressed in the “pre-metastatic” lung microenvironment could support the newly immigrant tumor cells by providing growth and angiogenic factors. Macrophages are known to play an important role in tumor growth by releasing pro-angiogenic molecules. Using mouse mammary tumor models, we have previously shown that during neoplastic progression both the mammary tumor cells and splenic macrophages from tumor-bearing mice express higher levels of CHI3L1 compared to normal control mice. However, the role of CHI3L1 in inducing angiogenesis by macrophages at the pulmonary microenvironment to support newly arriving breast cancer cells is not yet known. In this study, we determined the expression of CHI3L1 in bronchoalveolar lavage macrophages and interstitial macrophages in regulating angiogenesis that could support the growth of newly immigrant mammary tumor cells into the lung. Here we show that *in vitro* treatment of pulmonary macrophages with recombinant murine CHI3L1 resulted in enhanced expression of pro-angiogenic molecules including CCL2, CXCL2, and MMP-9. We and others have previously shown that inhibition of CHI3L1 decreases the production of angiogenic molecules. In this study, we explored if *in vivo* administration of chitin microparticles has an effect on the expression of CHI3L1 and pro-angiogenic molecules in the lungs of mammary tumor-bearing mice. We show that treatment with chitin microparticles decreases the expression of CHI3L1 and pro-angiogenic molecules in the “metastatic” lung. These studies suggest that targeting CHI3L1 may serve as a potential therapeutic agent to inhibit angiogenesis and thus possibly tumor growth and metastasis.

## Introduction

Metastasis is the major cause of death in breast cancer patients. It is well established that breast cancer often metastasizes to the lung. Preferential colonization of specific tissues by breast cancer cells could be partially determined by the nature of microenvironment within the target organ (Steeg, 2006). Recent studies have implicated specific cellular elements in the lungs that contribute to tumor growth. These include airway epithelial cells and immune cells, such as interstitial and alveolar macrophages, among others. Studies have demonstrated functional, morphological, and phenotypic differences between these interstitial and alveolar macrophages (Sebring and Lehnert, 1992; Prokhorova et al., 1994; Johansson et al., 1997). However, there are limited studies on interstitial macrophages in human lungs compared to alveolar macrophages, which can be easily obtained by bronchoalveolar lavage (BAL). The role of either interstitial or alveolar macrophages in the pre-metastatic lung in breast cancer metastasis has not yet been elucidated. We hypothesize that interstitial macrophages, alveolar macrophages, or both, may alter the pre-metastatic landscape of the lung.

Pulmonary macrophages may exert anti-tumor effects to suppress the growth of newly-immigrated breast cancer cells, or alternatively exert pro-tumor effects by producing growth factors that support their establishment in the lung. By release of proteases, growth factors and cytokines, activated macrophages have the potential to influence each phase of the angiogenic process. This includes stimulating remodeling of the local extracellular matrix, inducing endothelial cells to migrate or proliferate, and inhibiting formation of differentiated capillaries.

We and others have recently shown that a glycoprotein known as chitinase-3-like-1 protein is produced by macrophages from tumor-bearing hosts (Kawada et al., 2012; Libreros et al., 2012). Chitinase-3-like-1 glycoprotein (aka BRP-39, YKL-40) is a secreted protein that is upregulated in various types of cancers, including breast (Johansen et al., 2003). This molecule is synthesized under inflammatory conditions, including bronchial asthma, inflammatory bowel disease, and cancer, but is not highly expressed under physiological conditions (Johansen et al., 2006; Mizoguchi, 2006; Chupp et al., 2007; Coffman, 2008; Lee et al., 2011; Libreros et al., 2013). CHI3L1 is a chitin-binding glycoprotein that belongs to the family of chitinase-like proteins, but is lacking in enzymatic activity (Henrissat and Bairoch, 1993). This glycoprotein is expressed and secreted by a variety of cell types including articular chondrocytes, synoviocytes, osteoblasts, macrophages, neutrophils, and epithelial cells (Johansen et al., 2001; Rehli et al., 2003; Mizoguchi, 2006; Rathcke and Vestergaard, 2006; Lee et al., 2009). In examining the function of CHI3L1, we and others have found that CHI3L1 stimulates the production of pro-angiogenic molecules (Shao et al., 2009; Kawada et al., 2012; Libreros et al., 2012). Conversely, (Shao et al., 2009) and (Libreros et al., 2012), have shown that inhibiting CHI3L1 with neutralizing antibodies or administration of chitin microparticles, decreases the expression of pro-angiogenic molecules (Shao et al., 2009; Libreros et al., 2012).

Currently there is little known regarding mechanistic links between CHI3L1 expression by macrophages in the “pre-metatastic” lung, and tumor-related angiogenesis. We therefore examined the possibility that macrophage-derived CHI3L1 in the lung, upregulated by exposure to circulating CHI3L1 produced by tumor cells, “conditions” this organ to favor establishment of newly-arrived, metastasizing cancer cells. Thus, in this study we tested the role of CHI3L1 expression by bronchoalveolar and interstitial macrophages in regulating angiogenesis to promote the growth of new mammary tumor cells in the lung. We demonstrate here that: (1) CHI3L1 is secreted by interstitial and alveolar macrophages from mammary tumor bearers; (2) CHI3L1 induces expression of pro-angiogenic molecules in interstitial and alveolar macrophages; (3) *in vivo* treatment with chitin microparticles reduces angiogenesis; and (4) this treatment also decreases expression of CHI3L1, CCL2, CXCL2 and MMP-9. Our findings suggest that CHI3L1 may play a role in preparing the “soil” in the “pre-metastatic” lung. Therefore, CHI3L1 may be an attractive therapeutic target to inhibit breast cancer metastasis.

## Materials and methods

### Mice and cell lines

Female BALB/c mice were used in all studies (Charles River Laboratories, 8–12 week-olds), and were housed and used according to the National Institutes of Health guidelines, under protocols approved by Florida Atlantic University Institutional Animal Care and Use Committee. Mammary tumor cells (4T1-luc-A4; Caliper Life Sciences, Hopkinton, MA) were maintained in RPMI with 10% FCS (Kim et al., 2010). Tumor cells were implanted in mice by subcutaneous injection of 1 × 10^5^ 4T1 tumor cells in the lower right ventral quadrant. These tumors metastasize to the lung ~4–5 weeks post-tumor cell implantation. Normal mice and tumor bearers were assessed at ~2 weeks- (“pre-metastatic”) and 5 weeks post-tumor implantation (“metastatic”) for the expression of cytokines. Tumor bearers treated with chitin microparticles (see below) were assessed at 5 weeks post-tumor cell implantation for metastasis, cytokine expression and tumor angiogenesis using AngioSense probe.

### Isolation of epithelial cells and macrophages from the lungs

#### Bronchoalveolar macrophage isolation

Tracheas of euthanized mice were cannulated and lavaged with 1 mL of saline. The bronchoalveolar lavage fluid (BALF) was recovered to obtain alveolar macrophages as described by Kogiso et al. (2011). Plastic-adherent macrophages were then enriched by incubating BALF cells (10^6^ cells/mL) in complete media (RPMI 1640 with 10% FBS, 10 units/mL penicillin and 10 μg/mL streptomycin) for 1 h in a 5% CO_2_ incubator at 37°C. Non-adherent cells were removed by washing with media, and the BALF macrophages were then isolated by labeling with CD11b magnetic microbeads (Miltenyi Biotec, Cambridge, MA). CD11b^+^ cells were purified by positive selection using AutoMACs (Miltenyi). Alveolar CD11b^+^macrophages isolated from all animals revealed >90% purity as determined by flow cytometric analysis (FACS Calibur).

#### Isolation of alveolar epithelial cells and interstitial macrophages

Immediately after collecting BALF specimens, pulmonary epithelial cells and interstitial macrophages were isolated as previously described, with modifications [35]. Media (1 mL, RPMI 1640 with 2% FBS and 1 mg/mL dispase (Invitrogen, Grand Island, NY)) was perfused into the airways and alveoli of the lungs. The lungs were then removed and incubated at 37°C for 1 h in the same media with 2 mg/mL collagenase type II (Invitrogen) added, followed by tissue mincing. Cells were filtered through Teflon mesh of 40 μM pores and washed with the media with no centrifugation at 0°C. Macrophages were isolated by plastic adherence, and were then purified by magnetic beads as described. The purity of the cells was >90% for CD11b expression as determined by flow cytometric analysis. Non-adherent cells (alveolar epithelial cells) were labeled with CD146 microbeads (MiltenyiBiotec) and purified by AutoMACs (MiltenyiBiotec). The purity was confirmed to be >90% as assessed by flow cytometric analysis.

#### Cell culture

Purified alveolar epithelial cells, and alveolar and interstitial macrophages, were cultured at 2 × 10^6^ cells/mL for 18 h in complete media as described previously (Owen et al., 2005). All cells were stimulated with either 500 ng/mL LPS (Sigma Chemical Co., St. Louis, MO), or in combination with 1 ng/mL or 5 ng/mL of endotoxin-free rmCHI3L1 (R&D systems, Minneapolis, MN). Growth media (GM) was included as a control condition for some of the cultures. Cell-free supernatants were then collected and stored at −80°C.

### Flow cytometry

Total lung homogenates and BALF cells were isolated and resuspended in FACS buffer (PBS with 1% BSA and 0.1% sodium azide) and stained for 30 min at 4°C with FITC-conjugated antibodies against CD11b (BDBiosciences, San Jose, CA), APC-conjugated Ly6C, PerCP-conjugated Ly6G, PerCPF4/80 (all from BDBiosciences) or CD146 (Miltenyi Biotec). Isotype-matched IgG was used for internal controls. For intracellular cytokine staining, BD cytofix/cytoperm + fixation/permeabilization kit were used according to manufacturer's instructions and this was followed by labeling for CHI3L1. CHI3L1 antibody (generously provided by Dr. Alison Humbles, MedImmune, MD) was conjugated to a fluorescent dye using Lightning-Link PE conjugation kit (Novus, Littleton, CO) following manufacturer's instructions. Samples were acquired in a FACSCalibur flow cytometer (BD Biosciences) and analyzed by Flow Jo software (Tree Star, Inc., Ashland, OR).

### Cytokine ELISA

BALF samples and culture supernatants from cells obtained from control and mammary tumor bearers were analyzed for CHI3L1, CCL2, CXCL2 and MMP-9 levels by ELISA (all from R&D Systems) according to manufacturer's instructions. Absorbance at 450 nm with wavelength correction at 570 nm was measured with a Tecan SLT Rainbow Reader (Lab Instruments, Research Triangle Park, NC) and optical density (OD) values of samples were converted to picograms against a standard curve plotted from known quantities of recombinant murine cytokines.

### Western blot analysis

Cells from total lung homogenates were lysed with sample buffer (20 mM dithiothreitol, 6% SDS, 0.25 M Tris, pH 6.8, 10% glycerol, 10 mM NaF and bromophenyl blue) and used to extract total protein. Total protein (20 μg) was resolved on 4–20% Mini-Protean SDS-PAGE gradient gels (BioRad Life Sciences, Hercules, CA) and transferred to PVDF membrane (Pierce) using a semi-dry transfer transblotter (BioRad). The membranes were blocked overnight at 4°C in SeaBlock (Calbiochem), and subsequently incubated at room temperature for 1 h with anti-mouse CHI3L1 polyclonal antibody (1 μg/mL) (Quidel, San Diego, CA) and anti-mouse beta actin polyclonal antibody (0.25 μg/mL) (Li-Cor Biosciences, Lincoln, NE). Immunoblots were washed with 0.5% Tween-PBS followed by 1 h incubation at room temperature with appropriate infrared dye-conjugated secondary antibodies (Li-Cor Biosciences). Blots were washed again with 0.5% Tween-PBS and then dried at 37°C for 20 min. The membranes then were imaged and bands quantified using the Li-Cor Odyssey imaging system. Levels of proteins of interest were normalized to beta-actin.

### Confocal microscopy

Lungs from control mice and 2-week mammary tumor bearers were perfused with a mixture of Optimal Tissue Cutting (OCT) embedding compound and PBS (70%/30% respectively). The lungs were then removed and snap frozen on dry ice. 5 μM cryostat sections were mounted on SuperFrost Plus slides (Fisher Scientific, Fair Lawn, NJ), fixed in 4% paraformaldehyde, and labeled with goat anti-mouse CC10 (1:100, Santa Cruz Biotech, Santa Cruz, CA) and rabbit anti-mouse CHI3L1 (1 μg/μL, Quidel) as described below. Alveolar and interstitial macrophages were plated (0.5 x 10^6^ cells) on coverslips, fixed in 4% paraformaldehyde, and labeled with goat anti-mouse CD68 (1:100, macrophage marker) (Santa Cruz Biotech) and rabbit anti-mouse CHI3L1 (Quidel). Both cryostat sections and cells were blocked in 10% normal horse serum in PBS prior to staining with primary antibodies overnight at 4°C. After washing in PBS, cells and sections were incubated in the following secondary antibodies: FITC-donkey anti-rabbit and PE-donkey anti-goat (1:2000, both from Invitrogen, Life Technologies). To visualize nuclei, material was mounted with Vectashield containing DAPI (Vector Laboratories, Burlingame, CA), and examined by confocal microscopy (Carl Zeiss LSM 700, Microimaging, Thornwood, NY).

## *In vivo* treatment with chitin microparticles

Chitin microparticles (1–10 μM, kindly provided by Dr. Yoshimi Shibata, Florida Atlantic University, FL) were prepared as described previously (Shibata et al., 1997; Strong et al., 2002; Nishiyama et al., 2006). Tumor-bearing mice were treated by intraperitoneal injection with chitin microparticles (1 mg/mouse) starting 3 days post-tumor implantation and continuing every third day for 5 weeks (Libreros et al., 2012).

## Angiogenesis determination

To assess vascularization *in vivo*, the near-infrared blood pool agent AngioSense 680 probe (2 nmol/mouse in 150 μL volume; Perkin Elmer, Waltham, MA) was injected via tail vein 24 h before imaging. Mice were imaged using a bioluminescence optical imager (IVIS Lumina LTE, Perkin Elmer). Maximal near infrared signals were quantified using Living Image 2.5 image analysis software (Xenogen, Perkin Elmer). Infrared signals are reported as photons/sec.

## Statistical analyses

Results are expressed as group means ± *SD*. Statistical analyses were performed using GraphPad Prism 3 software (LaJolla, CA). Statistical comparisons of paired groups were determined by Student's *t* tests. Values of *p* < 0.05 were considered statistically significant.

## Results

### CHI3L1 expression is increased in BALF and total lung from mammary tumor-bearing mice

Increased levels of CHI3L1 in the sera of breast cancer patients are associated with poor prognosis (Johansen et al., 2003). We have previously reported higher circulating levels of CHI3L1 in 4T1 mammary tumor-bearing mice compared with normal mice (Libreros et al., 2012). This tumor model shares similar characteristics with human breast cancer patients as mice bearing 4T1 mammary tumors exhibit spontaneous tumor cell metastasis to the lung. The levels of CHI3L1 in the lungs are increased during pulmonary inflammation, and inflammation is known to contribute to tumor growth and metastasis. Since it is known that breast tumor cells metastasize to the lung, we determined if CHI3L1 expression is specifically altered in lungs of mammary tumor bearers compared to control mice.

**Figure 1:**
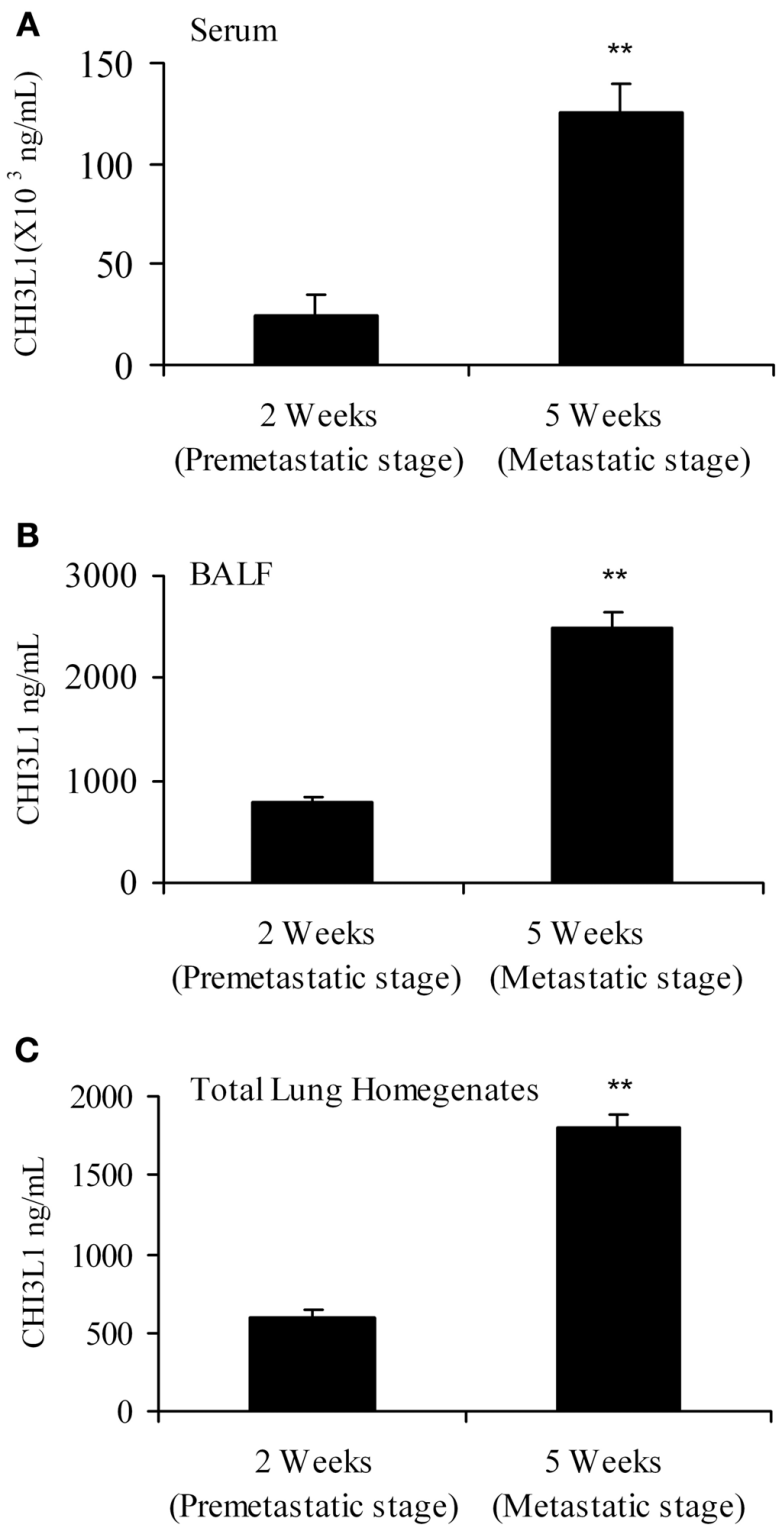
**CHI3L1 is expressed in serum and pulmonary tissue of mammary tumor-bearing mice at 2 weeks (“pre-metastatic”) and 5 weeks (metastatic) post-tumor cell implantation. (A)** Serum **(B)** BALF and **(C)** Total lung homogenates from 2 week and 5 week mammary tumor bearers were analyzed for CHI3L1 expression by ELISA. *N* = 10 mice/group; ^**^p ≤ 0.001.

**Figure 2:**
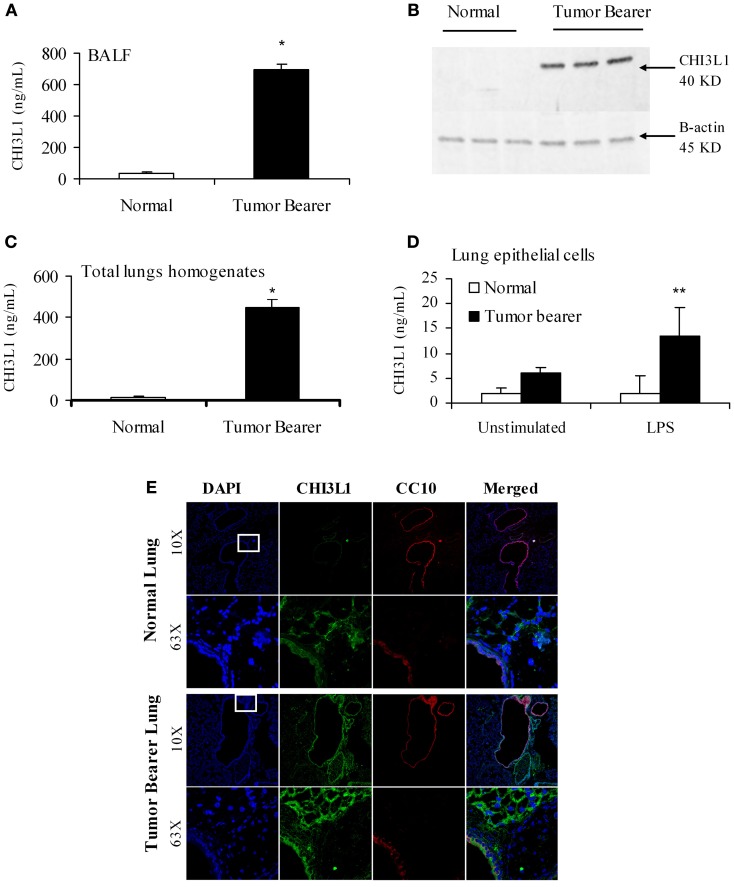
**CHI3L1 is expressed at higher levels in the pulmonary tissue of 2-week 4T1 mammary tumor bearers. (A)** CHI3L1 expression in bronchoalveolar lavage fluid by ELISA; **(B)** Western blot analysis of total lung homogenates for CHI3L1 expression; (**C,D**) CHI3L1 expression by ELISA of total lung homogenates **(C)** and lung epithelial cells **(D)**. **(E)** Cellular co-localization of CHI3L1 with CC10, an airway-epithelial cell marker, in cryostat sections visualized by confocal microscopy. For all experiments, *N* = 10/group; ^*^p ≤ 0.05; ^**^p ≤ 0.001.

The “pre-metastatic” and “metastatic” stages were described by Yan et al. using the 4T1 mammary tumor model, with the pre-metastatic stage occurring at 14 days post-tumor cell inoculation, and the metastatic stage at 4 weeks (Yan et al., 2010). We therefore assessed CHI3L1 expression in the lungs of mice inoculated with 4T1 mammary tumor cells at 2 weeks post-cell implantation, a time point at which no visible micro-metastasis is observed in the lungs (data not shown), and at 5 weeks when metastasis of 4T1 cells is known to be well-established (Yan et al., 2010; Libreros et al., 2012). We first measured circulating levels of CHI3L1, which increased from 25 × 10^3^ ng/mL at 2 weeks, to 125 × 10^3^ ng/mL at 5 weeks (Figure 1A). ELISA measurements demonstrated that significantly higher levels of CHI3L1 were also present in both BALF samples (Figure 1B) and total lung homogenates (Figure 1C) at 5 weeks post-tumor cell implantation, compared to the 2-week time point. These higher levels of CHI3L1 could be due to expression by the pulmonary tissue itself and/or the tumor cells that have infiltrated by 5 weeks (Libreros et al., 2013). Samples from the “pre-metastatic” stage would help differentiate between these possibilities, as tumor cells have not yet infiltrated, and we performed additional analyses at this stage. At 2 weeks post-inoculation, significantly higher levels of CHI3L1 were measured by ELISA in BALF samples from tumor bearers compared to control mice (Figure 2A). Western blot analysis of whole lungs from pre-metastatic tumor-bearers confirmed higher levels of pulmonary CHI3L1 (Figure 2B), and ELISA assays of total lung homogenates quantified this increase at 2 weeks (Figure 2C). CHI3L1 is secreted by a variety of cell types, including macrophages, neutrophils, colonic epithelial cells, and chondrocytes (Nyirkos and Golds, 1990; Hakala et al., 1993; Renkema et al., 1998; Volck et al., 1998; Mizoguchi, 2006), and recent studies by Lee et al. (2009) have shown that CHI3L1 (aka BRP-39) is upregulated in inflamed airway epithelium, and that it plays an active role in pulmonary inflammation (Lee et al., 2009). We therefore determined if CHI3L1 expression is specifically altered in lung epithelial cells isolated from mammary tumor bearers at 2 weeks post-inoculation, compared to those from control mice. Production of CHI3L1 was increased more than 5-fold in pulmonary epithelial cells from tumor bearers, as measured by ELISA at 18 h post-plating (Figure 2D). To promote “inflammatory” conditions, cultures were treated with LPS to stimulate cytokine production, which exacerbated the increase in CHI3L1 levels displayed by cells from tumor bearers (Figure 2D). Localization of CHI3L1 in lung tissue samples by immunofluorescence showed that CHI3L1 was expressed by lung epithelial cells (CC10^+^ cells), and that this expression was increased in the airways of mammary tumor bearing mice compared to controls (Figure 2E).

### CHI3L1 expression is increased in CD11b^+^Gr1^+^ cells of mammary tumor bearers

**Figure 3:**
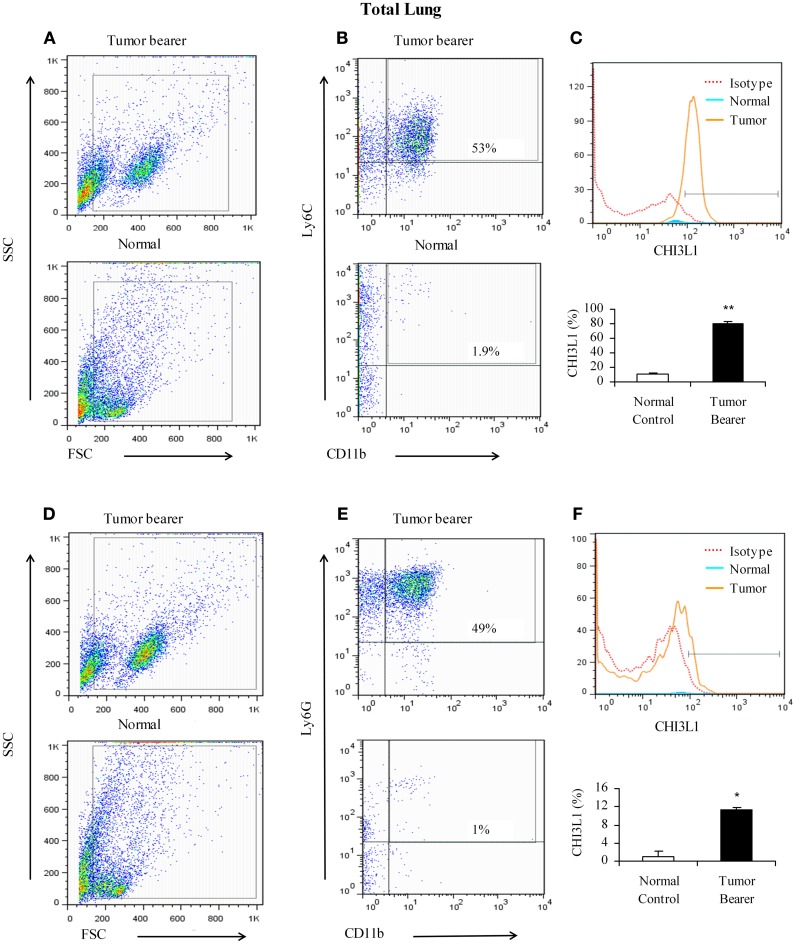
**CHI3L1 is expressed at higher levels in CD11b^+^Ly6C^+^ total lung cells in 2-week mammary tumor bearers. (A,D)** Representative scatter plots of forward scatter vs. side scatter in total lung from normal and tumor bearers; **(B)** CD11b^+^Ly6C^+^ cells from total lungs of normal and tumor bearers and assessed for CHI3L1 expression; **(C)** Representative overlay histogram plot gated on CD11b^+^ and Ly6C^+^ cells for CHI3L1 expression; **(E)** CD11b^+^Ly6G^+^ cells from total lungs of normal and tumor bearers and assessed for CHI3L1 expression; **(F)** Representative overlay histogram plot gated on CD11b^+^ and Ly6G^+^ cells for CHI3L1 expression. For all experiments, *N* = 10/group; ^*^p ≤ 0.05; ^**^p ≤ 0.001.

Myeloid-derived cells have been shown to be important in promoting tumor growth, metastasis, and angiogenesis (van Kempen and Coussens, 2002; Yang et al., 2004). The lungs of 4T1 mammary tumor bearers show infiltration by myeloid-derived suppressor cells, and in particular by CD11b^+^Gr1^+^ cells that establish a pre-metastatic niche by secreting proinflammatory mediators (Yan et al., 2010; Younos et al., 2011). We have previously shown that splenic myeloid cells from mammary tumor-bearing mice express CHI3L1 (Libreros et al., 2012). To clearly delineate myeloid populations of cells in pulmonary tissue that could contribute to CHI3L1 expression, single cell suspensions prepared from total lungs of normal and 2-week mammary tumor-bearers were analyzed by flow cytometry. As shown in Figures 3A–C (one representative assay out of five), CD11b^+^Ly6C^+^ cells from mammary tumor bearers express CHI3L1 at higher levels compared to normals. CD11b^+^Ly6G^+^ cells from tumor bearers express CHI3L1 but these levels are lower compared to the levels observed in CD11b^+^Ly6C^+^ cells (Figures 3D–F).

**Figure 4:**
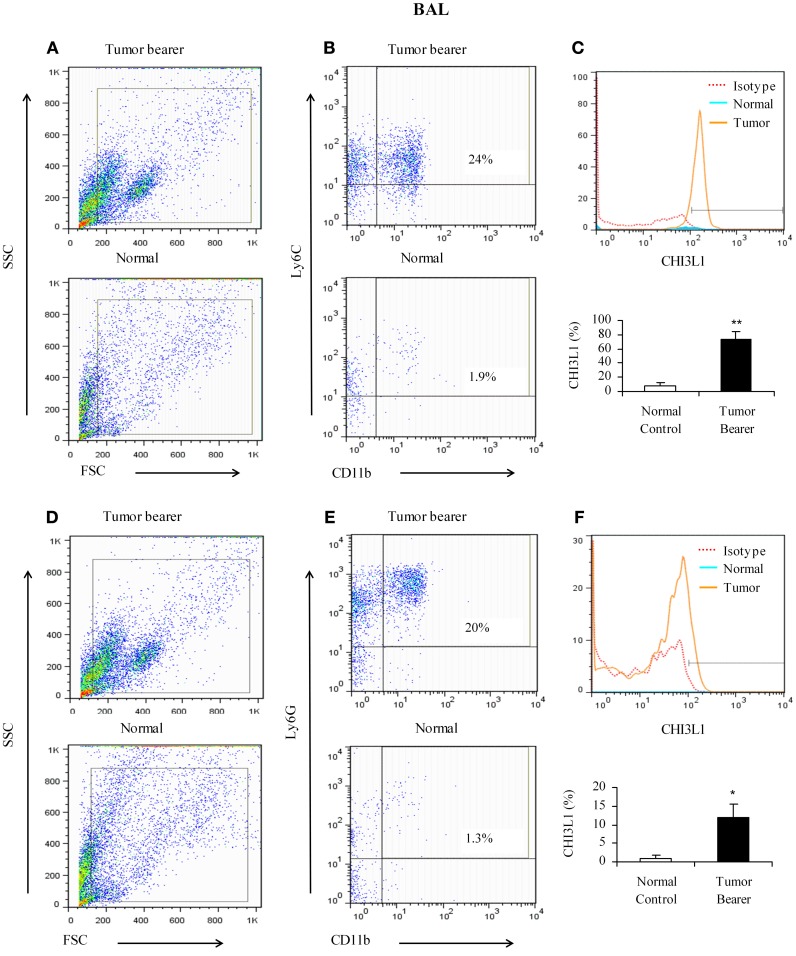
**CHI3L1 is expressed at higher levels in CD11b^+^Ly6C^+^ bronchoalveolar lavage cells in 2-week mammary tumor bearers. (A,D)** Representative scatter plots of forward scatter vs. side scatter in alveolar lavage from normal and tumor bearers; **(B)** CD11b^+^Ly6C^+^ cells from alveolar lavage of normal and tumor bearers and assessed for CHI3L1 expression; **(C)** Representative overlay histogram plot gated on CD11b^+^ Ly6C^+^ cells for CHI3L1 expression; **(E)** CD11b^+^Ly6G^+^ cells from alveolar lavage of normal and tumor bearers and assessed for CHI3L1 expression; **(F)** Representative overlay histogram plot gated on CD11b^+^Ly6G^+^ cells for CHI3L1 expression. For all experiments, *N* = 10/group; ^*^p ≤ 0.05; ^**^p ≤ 0.001.

Since BALF was shown to contain elevated levels of CHI3L1, we determined which cell populations in the lavage contribute to the expression of CHI3L1 at 2 weeks post-tumor cell inoculation. Toward this we assessed CD11b^+^Ly6C^+^ and CD11b^+^Ly6G^+^ cells in the lavage. CD11b^+^Ly6C^+^ cells from mammary tumor bearers express higher levels of CHI3L1 compared to normals (Figures 4A–C). Similar to what was observed in total lung homogenates, CD11b^+^Ly6G^+^ cells from tumor bearers express CHI3L1 at lower levels compared to the CD11b^+^Ly6C^+^ cells (Figures 4D–F).

### CHI3L1 expression is increased in macrophages from the lungs of mammary tumor-bearing mice

**Figure 5:**
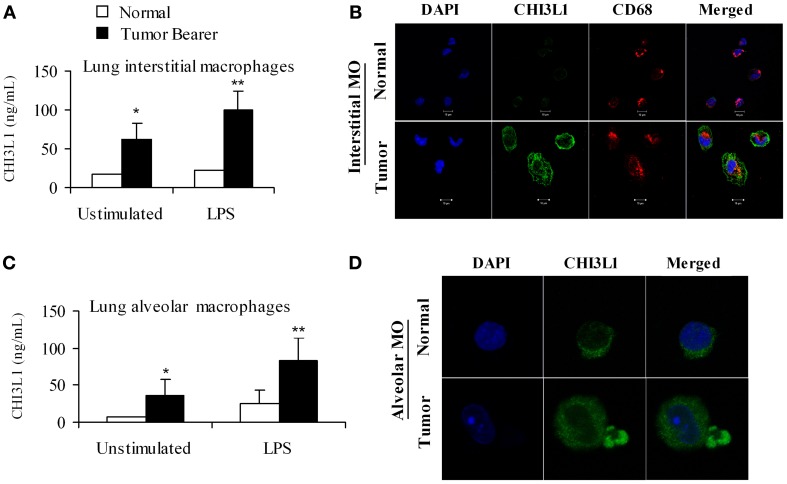
**CHI3L1 is expressed at higher levels in pulmonary macrophages from 2-week mammary tumor bearing mice. (A)** CHI3L1 ELISA of interstitial macrophages from normal and mammary tumor bearers; **(B)** Cellular co-localization of CHI3L1 with CD68, a macrophage marker was visualized by confocal microscopy, scale bar 10 μM; **(C)** CHI3L1 ELISA of alveolar macrophages from normal and mammary tumor bearers; **(D)** Cellular localization of CHI3L1 was visualized by confocal microscopy, scale bar 10 μM. For all experiments, *N* = 10/group; ^*^p ≤ 0.05; ^**^p < 0.001.

We have previously shown that CHI3L1 is expressed at higher levels in splenic macrophages of mammary tumor-bearing mice (Libreros et al., 2012). In this study, we determined the expression levels of CHI3L1 in macrophages from the “pre-metastatic” lungs. Two broad subsets of macrophages are found in the lungs of mice and humans, i.e., alveolar macrophages which line the surface of alveoli, and interstitial macrophages that are localized in the space between alveolar epithelium and vascular endothelium (Schneberger et al., 2011). Thus, alveolar and interstitial macrophages from normal and 2-week tumor bearers were purified as described in the Methods section, and cultured in either the absence or presence of LPS. Interstitial macrophages from mammary tumor-bearing mice secrete CHI3L1 and these levels were further increased by stimulation with LPS as determined by ELISA (Figure 5A). Localization of CHI3L1 in interstitial macrophages was then confirmed by immunofluorescent labeling. Confocal images revealed higher intensity of CHI3L1 expression in CD68^+^interstitial macrophages from tumor bearers, relative to normal mice (Figure 5B). Purified alveolar macrophages were also analyzed. Similar to what was observed in the interstitial macrophage population, there were higher than normal levels of CHI3L1 present in culture supernatants of alveolar macrophages from 2 week tumor-bearing mice (Figure 5C). Intensity of CHI3L1 staining in alveolar macrophages similarly was greater in tumor bearers' macrophages, as determined by confocal microscopy (Figure 5D). Interestingly, the expression levels of CHI3L1 were higher in interstitial macrophages compared to alveolar macrophages.

### CHI3L1 exacerbates the production of pro-angiogenic molecules in LPS-stimulated pulmonary macrophages

**Figure 6:**
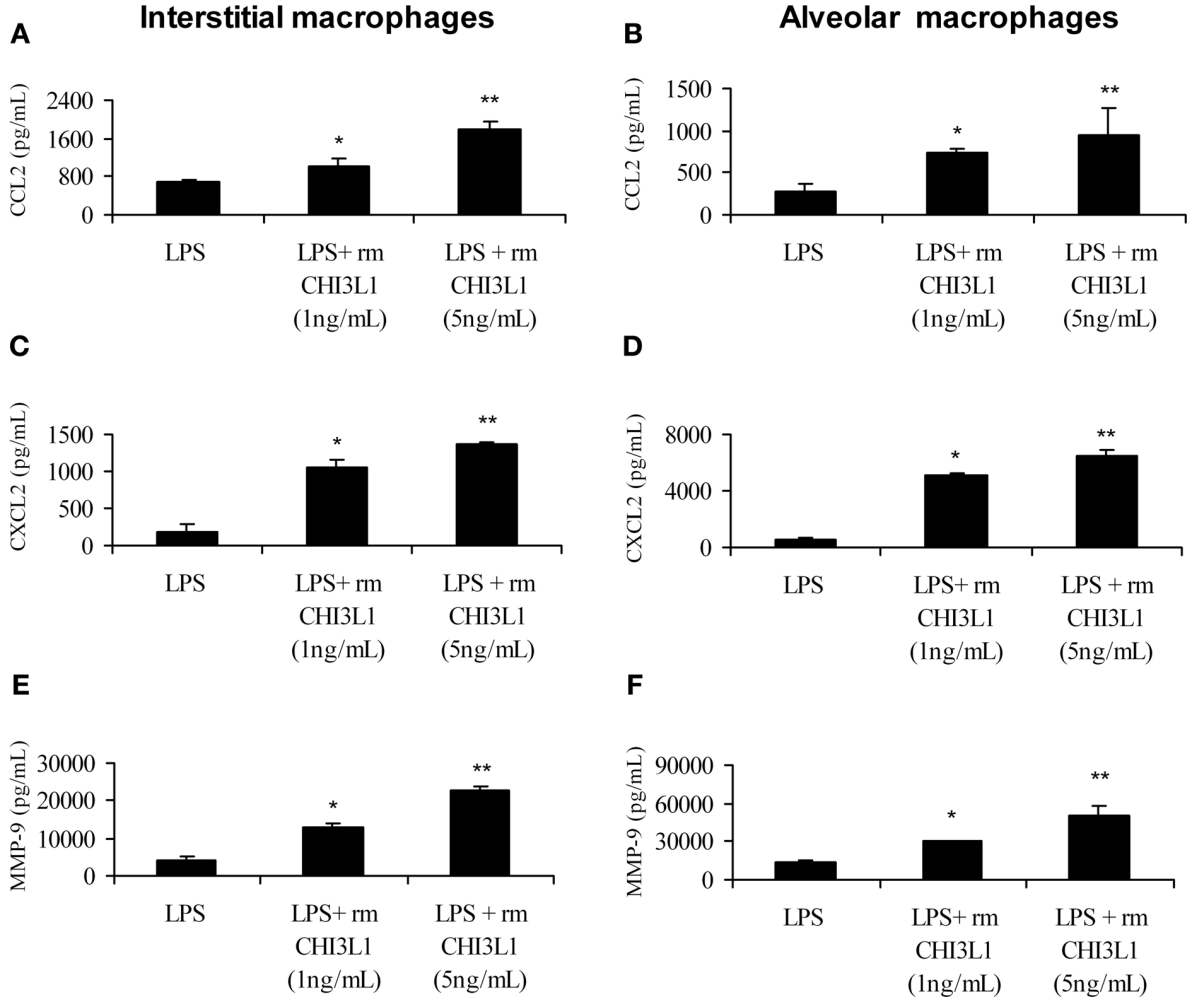
**CHI3L1 increases the expression of CCL2, CXCL2 and MMP-9.** Pulmonary macrophages from normal mice were cultured overnight with LPS (500 ng/mL) alone and with either 1 ng/mL or 5 ng/mL of rmCHI3L1. Cell free supernatants were then analyzed for CCL2 in: **(A)** interstitial or **(B)** alveolar macrophages; CXCL2 in: **(C)** interstitial or **(D)** alveolar macrophages; and MMP-9 in: **(E)** interstitial or **(F)** alveolar macrophages. For all experiments, *N* = 10/group; ^*^p ≤ 0.05; ^**^p ≤ 0.001.

Yan et al. (2010) found that the expression of MMP-9 and CCL-2 is upregulated in the lungs of 2 week tumor-bearing mice compared to normal mice (Yan et al., 2010). We and others have shown that CHI3L1 induces the expression of CCL2, CXCL2 and MMP-9 in splenic macrophages (Mizoguchi, 2006; Letuve et al., 2008; Kawada et al., 2012; Libreros et al., 2012), but there are few studies to date on the biological role of CHI3L1 in pulmonary macrophages. In this study we tested the effects of CHI3L1 on interstitial and alveolar macrophages isolated from normal mice, and analyzed the production of the pro-angiogenic molecules CCL2, CXCL2 and MMP-9 by ELISA. Cells were treated with CHI3L1 in combination with LPS, which is necessary for expression of angiogenic molecules by *ex vivo* macrophages. Treatment of either interstitial or alveolar macrophages with LPS alone or in combination with rmCHI3L1 (1 ng/mL or 5 ng/mL) resulted in a dose-dependent increase in the production of CCL2 (Figures 6A,B), CXCL2 (Figures 6C,D) and MMP-9 (Figures 6E,F) in both interstitial and alveolar macrophages. Culturing with rmCHI3L1 alone in the absence of LPS revealed a similar trend to the one observed in cultures containing both LPS and rmCHI3L1, but the levels of the proinflammatory mediators secreted were lower (data not shown). The combined effects of LPS and rmCHI3L1 produced the greatest increase in the expression of pro-angiogenic molecules.

### *In vivo* treatment with chitin microparticles decreases angiogenesis

**Figure 7:**
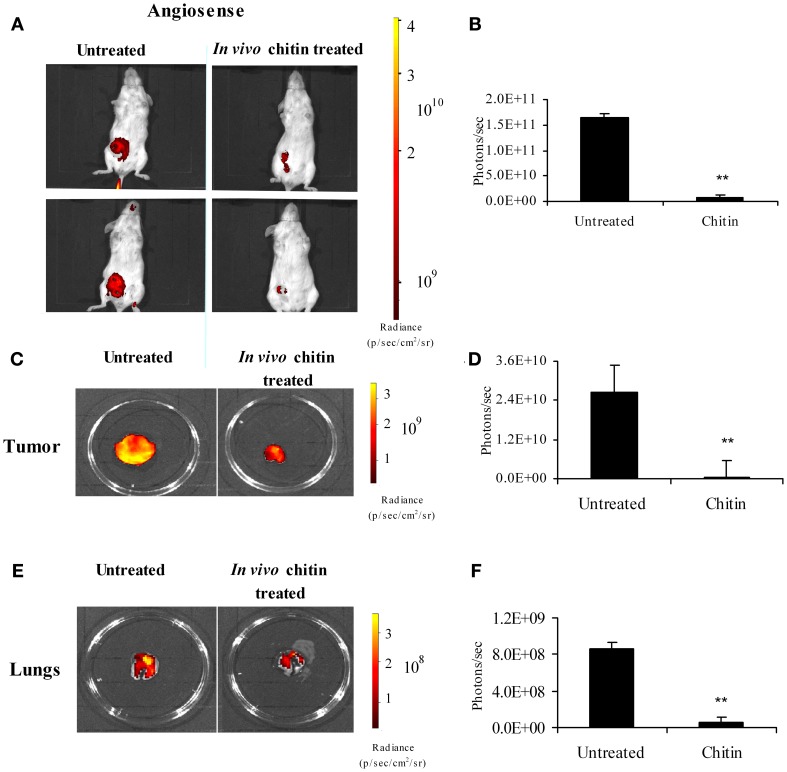
***In vivo* treatment with chitin microparticles decreases angiogenesis.** 4T1 mammary tumor bearers at 5 weeks post-tumor implantation were treated *in vivo* with chitin microparticles (1mg/mL) and analyzed for angiogenesis using AngioSense probe by *in vivo* imaging system. **(A)** Ventral image of untreated and chitin treated mammary tumor bearing mice showing 2 out 5 mice/treatment group; **(B)** Quantification (photons/s) of AngioSense specific fluorescent signal indicating decreased angiogenesis at 21 days post-tumor implantation in the chitin treated group; **(C)** Excised tumors from untreated and chitin treated mice indicating decreased fluorescent signal in the chitin treated group; **(D)** Quantification (photons/s) of AngioSense specific fluorescent signal indicating decreased angiogenesis at 21 days post-tumor implantation in the chitin treated group; **(E)** Excised lungs from untreated and chitin treated mice indicating decreased fluorescent signal in the chitin treated group; **(F)** Quantification (photons/s) of AngioSense specific fluorescent signal indicating decreased angiogenesis in the lungs at 21 days post-tumor implantation in the chitin treated group compared to untreated group. For all experiments, *N* = 5/group; ^**^p ≤ 0.001.

Intraperitoneal treatment of mammary tumor bearers with chitin microparticles, a substrate for CHI3L1, results in decreased tumor growth and pulmonary metastasis (Libreros et al., 2012). We and others established that 4T1 mammary tumors begin to infiltrate the lungs by 3 weeks post-tumor cell implantation, and observable metastatic foci are seen in the lungs at 5 weeks post-tumor cell implantation (Yan et al., 2010; Libreros et al., 2012). Therefore, we used 5 week tumor bearers to assess the effects of *in vivo* chitin microparticle treatment. To test if early treatment with chitin microparticles affects angiogenesis and tumor growth, an *in vivo* AngioSense probe was used. Mice treated with chitin microparticles after tumor cell inoculation had significantly reduced angiogenic fluorescent signals in *in vivo* imaged tumors compared to the tumors from untreated mice (Figure 7A). Quantification of fluorescent signals confirmed these results (Figure 7B). Previously we noted that tumor size in chitin-treated, 5 week tumor-bearing mice was significantly smaller than normal. We now show with the AngioSense probe that there was a significant decrease in angiogenic fluorescent signals in the excised tumors of chitin-treated mice, compared to untreated tumor bearers (Figure 7C). Quantification of fluorescent signals is depicted in Figure 7D.

Following metastasis, decreased tumor growth in the target organ also could be due to decreased angiogenesis. Since treatment with chitin microparticles decreased angiogenesis in the primary tumor, we tested to see if *in vivo* treatment with chitin microparticles also had an effect on angiogenesis in the metastatic lung. Excised lungs from chitin-treated and untreated mice at 5 weeks post-tumor cell inoculation were imaged as above to evaluate angiogenesis. Similar to what was observed in the tumor tissue, there was a significant reduction in fluorescent AngioSense signals, indicating decreased angiogenesis in the lungs of mice treated with chitin microparticles, compared to untreated controls (Figure 7E). Quantification of these fluorescent signals is shown in Figure 7F.

### *In vivo* treatment with chitin microparticles decreases the production of pro-angiogenic molecules by pulmonary macrophages

**Figure 8:**
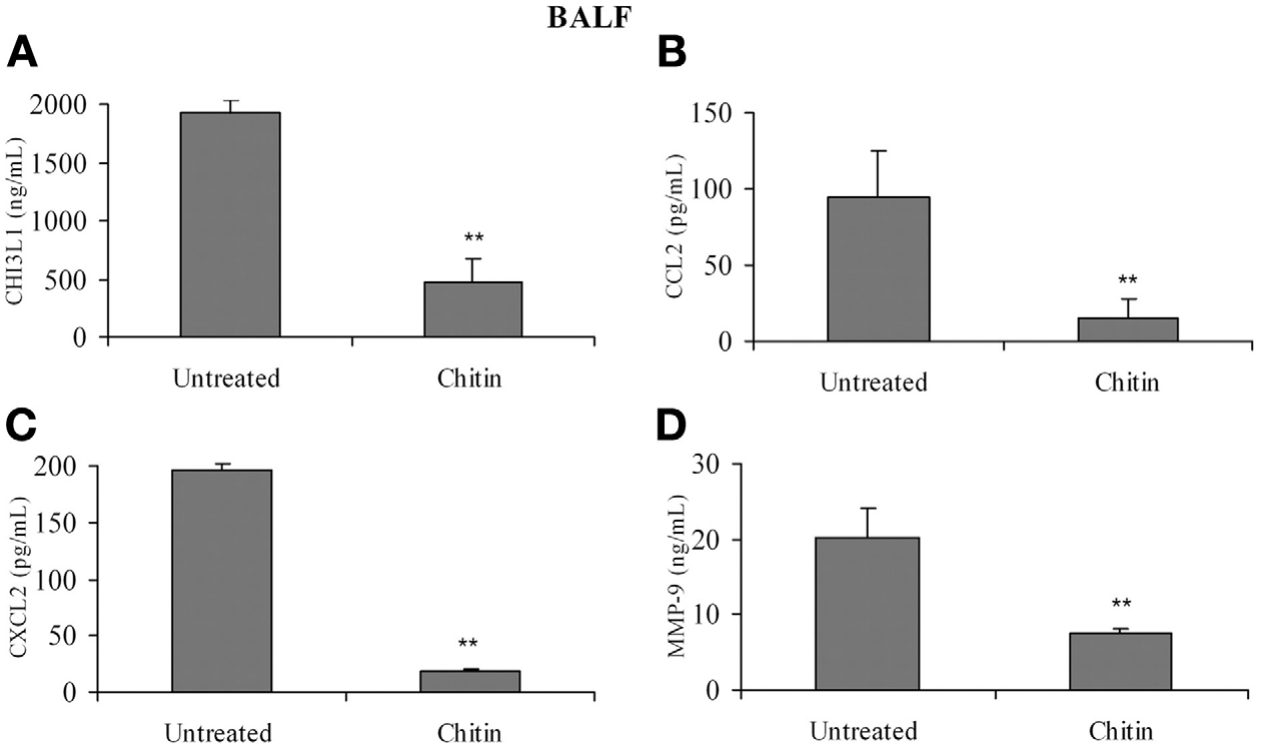
***In vivo* treatment with chitin microparticles decreases CHI3L1, CCL2, CXCL2, and MMP-9 expression in BALF.** BALF from untreated and chitin treated mice at 5 weeks post-tumor implantation was analyzed for: **(A)** CHI3L1; **(B)** CCL2; **(C)** CXCL2; and **(D)** MMP-9 expression by ELISA. For all experiments, *N* = 10/group; ^**^p ≤ 0.001.

**Figure 9:**
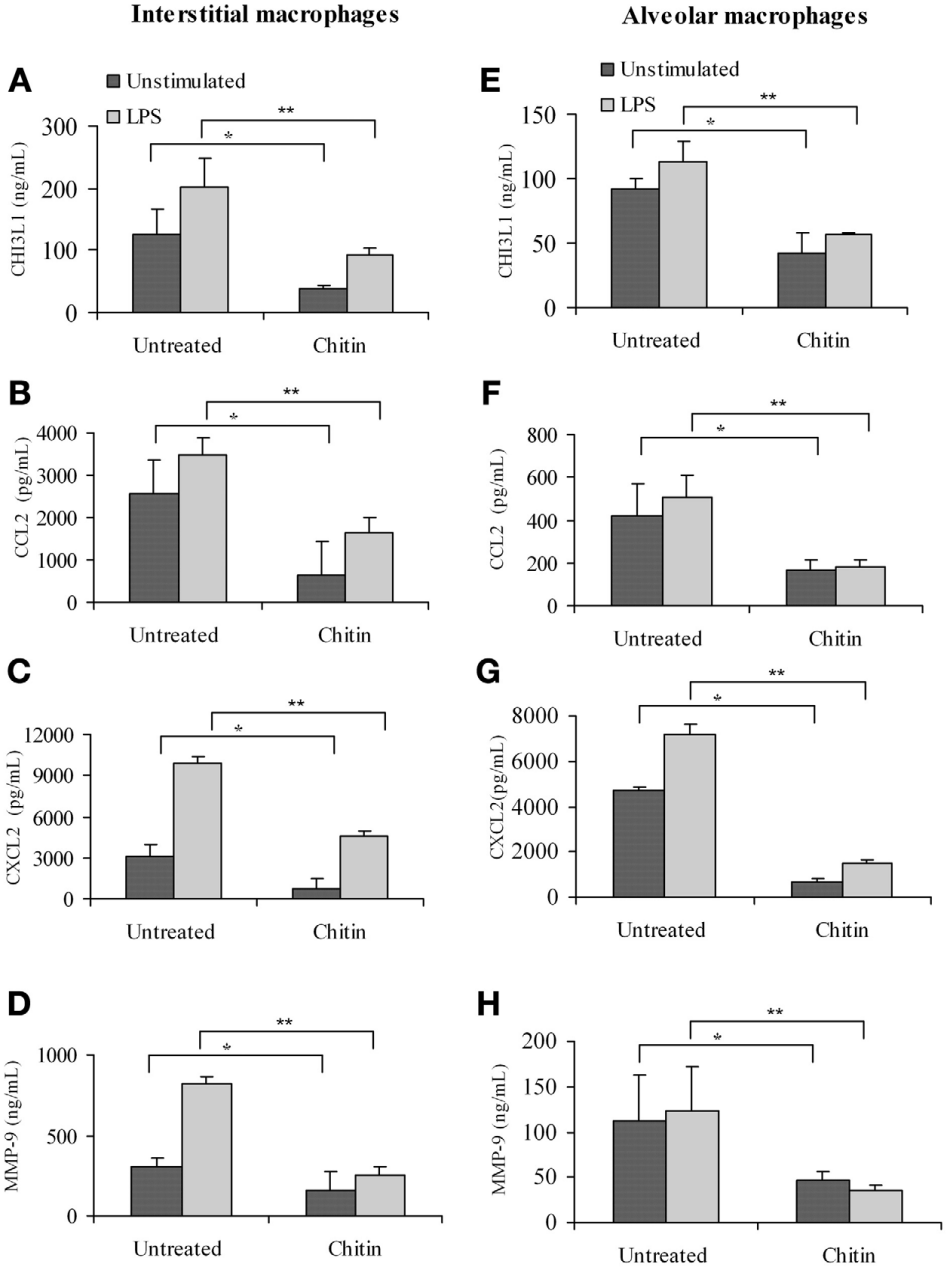
***In vivo* treatment with chitin microparticles decreases CHI3L1, CCL2, CXCL2, and MMP-9 expression in pulmonary macrophages from 5 week mammary tumor bearers.** Interstitial macrophages from untreated and chitin treated mice was analyzed for: **(A)** CHI3L1; **(B)** CCL2; **(C)** CXCL2; and **(D)** MMP-9 expression by ELISA, and alveolar macrophages from untreated and chitin treated mice was analyzed for: **(E)** CHI3L1; **(F)** CCL2; **(G)** CXCL2; and **(H)** MMP-9 expression by ELISA. For all experiments, *N* = 10/group; ^*^p ≤ 0.05; ^**^p ≤ 0.001.

Faibish et al. (2011) found that neutralizing antibody to YKL-40 blocks tumor angiogenesis by inhibiting endothelial cell tube formation (Faibish et al., 2011) while (Kawada et al., 2012) demonstrated increased microvessel density in CHI3L1-transfected colon cancer cells (Kawada et al., 2012). Since CHI3L1 expression by splenic macrophages is altered *in vivo* by chitin microparticle treatment (Libreros et al., 2012) we next determined if CHI3L1 expression in lung tissue is similarly affected. Bronchoalveolar lavage fluid was analyzed for the expression of pro-angiogenic molecules. *In vivo* treatment with chitin microparticles resulted in decreased CHI3L1 expression in BALF collected form 5 week tumor-bearers (Figure 8A). As CHI3L1 promotes expression of pro-angiogenic molecules, we reasoned that decreased CHI3L1 levels in chitin-treated mice should result in decreased expression of pro-angiogenic molecules. We found that the levels of CCL2 (Figure 8B), CXCL2 (Figure 8C) and MMP-9 (Figure 8D) in BALF samples were indeed decreased by *in vivo* chitin treatment. We also found that interstitial and alveloar macrophages from chitin-treated mammary tumor bearers exhibited reduced expression of CHI3L1, and these same pro-angiogenic molecules, at 5 weeks post-tumor cell inoculation (Figure 9).

## Discussion

Circulating tumor cells must invade and proliferate in a target organ to establish metastasis. It is well established that preferential tissue colonization is determined not only by features intrinsic to the type of tumor cell, but also in part by the unique nature of each target organ (Steeg, 2006). The microenvironment, the “soil” or the “pre-metastatic niche,” in the target organ contributes to the survival of these cells. To our knowledge, there are no studies to date defining the role of CHI3L1 in pulmonary tissue in terms of promoting survival and growth of invading breast cancer cells. In this study, we examined the role of pulmonary macrophages in preparing the “soil” or the “pre-metastatic niche” for establishing breast cancer metastasis. We used an *in vivo* mouse mammary tumor model mimicking CHI3L1 expression in breast cancer patients to examine the role of CHI3L1 and CHI3L1-induced angiogenic molecules in the pulmonary microenvironment during the emergence of metastasis.

We show here that CHI3L1 levels are increased in both the “pre-metastatic” lung and “metastatic” lung of mammary tumor-bearing mice. Higher levels of CHI3L1 were observed not only in the serum, but also in BALF and lung tissue homogenates. We found that expression of CHI3L1 is upregulated in lung epithelial cells, as well as in alveolar and interstitial macrophages of mammary tumor-bearing mice. Importantly, CHI3L1 was found to induce the production of angiogenic molecules, CCL2, CXCL2 and MMP-9 in both alveolar and interstitial macrophages from normal mice. We also demonstrate that *in vivo* treatment with chitin microparticles, a substrate for CHI3L1, resulted in decreased production of CHI3L1, CCL2, CXCL2, and MMP-9 in BALF, and more specifically by interstitial and alveolar macrophages of mammary tumor-bearing mice. Decreased production of these molecules has been correlated with decreased levels of angiogenesis in tumors (Arenberg et al., 1998; Mehrad et al., 2007; Gerber et al., 2009). Transfection of HCT116 tumor cells with CHI3L1 enhances tumor growth, while *in vivo* treatment with anti-CHI3L1 neutralizing antibodies decreases angiogenesis (Shao et al., 2009; Kawada et al., 2012). Using administration of chitin microparticles, a molecule that binds to chitinases and chitin-like molecules (Ober and Chupp, 2009), we have shown previously that splenic macrophages from treated mice produce lower levels of pro-angiogenic molecules compared to untreated mammary tumor bearers, and that tumor growth and metastasis are reduced by this treatment (Libreros et al., 2012). To monitor angiogenesis during the “metastatic” stage, in this study we used the AngioSense 680 probe, a marker for blood vessel density, to directly assess *in vivo* tumor angiogenesis. *In vivo* imaging clearly demonstrated the effectiveness of chitin microparticle treatment on angiogenesis. Chitin-treated mice had lower fluorescent signals compared to the untreated controls, and more importantly, excised tumors, as well as lungs from treated mice, had lower levels of AngioSense signals compared to the untreated group. It is well-established that tumors do not grow >1 mm^3^ in size without an adequate blood supply (Folkman, 1971), and our results indicate that decreased angiogenesis in mammary tumors correlates well with the smaller size of these tumors in chitin-treated mice. In this study, tumor size as determined by assessing for luciferase signals revealed <0.5 × 10^6^ photons in treated group vs. 3 × 10^6^ in untreated group (*p* < 0.005). Reduced angiogenesis in the lungs also correlates well with the lower levels of pro-angiogenic molecules expressed by alveolar and interstitial macrophages in the chitin-treated group.

Macrophages have been described to be one of the key players in many types of cancers by producing a variety of factors that can either promote or inhibit tumor growth and metastasis (Mantovani et al., 2002). Numerous studies have reported on the role of tumor-infiltrating macrophages (TAMs) on tumor growth. There are very few reports on the role of macrophages at a metastatic site in terms of supporting the growth of infiltrating tumor cells. As the metastatic site offers new challenges for circulating tumor cells in terms of their survival, our focus has been to characterize macrophages in the lung microenvironment of mammary tumor bearers in terms of how they may support invading breast cancer cells. Although tumor cells and activated splenic macrophages express CHI3L1, a molecule associated with poor prognosis in breast cancer patients (Lal et al., 1999; Lau et al., 2006; Coffman, 2008; Libreros et al., 2012), few studies have analyzed CHI3L1 expression by alveolar and interstitial macrophages in tumor bearing models. In comparing the different cell types present in the lung, i.e., epithelial cells and macrophages, we found that both interstitial and alveolar macrophages from 2 week mammary tumor-bearing mice express much higher levels of CHI3L1 compared to epithelial cells. Prior work by Chupp et al., reported higher levels of CHI3L1 expression in biopsied lung tissue from patients with severe asthma, compared to those with a milder form, and that it localizes to the subepithelium of pulmonary tissue (Chupp et al., 2007). Using ova-sensitized and challenged mice, Lee et al. (2009) showed that CHI3L1 is expressed by airway epithelial cells and F4/80-positive macrophages during antigen-induced inflammation (Lee et al., 2009). Our results suggest that at early, pre-metastatic stages, CHI3L1 expression by either the airway epithelium or by “activated” lung macrophages may be induced by circulating tumor-derived factors including CHI3L1, and that this in turn promotes conditions that favor the later establishment of infiltrating tumor cells.

The biological roles of CHI3L1 have been recently characterized in terms of cell proliferation, angiogenesis, chemotaxis, and cell adhesion (Coffman, 2008; Shao et al., 2009; Kawada et al., 2012). We have previously shown that splenic macrophages from mammary tumor-bearing mice secrete higher levels of the pro-angiogenic molecules CCL2, CXCL2, and MMP-9 (compared to non-tumor bearers) and that CHI3L1 stimulates this increased production (Libreros et al., 2012). There are only few studies to date that have compared the production of angiogenic molecules by alveolar and interstitial macrophages in response to CHI3L1 in the context of inflammation. Letuve et al., showed that smokers with chronic obstructive pulmonary disease (COPD) had elevated serum levels of CHI3L1, and BALF samples contained a greater proportion of alveolar macrophages expressing CHI3L1 than smokers without COPD or non-smokers (Letuve et al., 2008). Inflammation associated with pulmonary sarcoid granulomas is also accompanied by expression of CHI3L1protein by both mononuclear cells/macrophages and giant cells of the granuloma (Johansen et al., 2005). Expression of CHI3L1 in the inflamed pulmonary environment may affect the function of local lung macrophages, and thereby favor the production of pro-angiogenic substances that promote tumor establishment and growth. Our evidence suggests that expression of CCL2, CXCL2, and MMP-9 by LPS-treated interstitial and alveolar macrophages from normal mice is enhanced by rmCHI3L1. These results are in agreement with those of Letuve et al., and Kawada et al., in that CHI3L1 stimulates macrophage production of IL-8 (homolog of mouse CXCL2), MCP-1 (CCL2) and MMP-9 (Letuve et al., 2008; Kawada et al., 2012). In addition to its angiogenic function, CCL2 acts as a chemoattractant molecule that recruits not only tumor cells, but also leukocytes that provide growth factors for the immigrant population of tumor cells (Carr et al., 1994; Craig and Loberg, 2006). We have previously shown that T lymphocytes from mammary tumor-bearing mice produce CCL2 and that T cell-derived CCL2 could also contribute to tumor growth directly via its pro-angiogenic activity and indirectly by attracting monocytes that secrete growth-promoting factors (Owen et al., 2005). Decreased levels of CCL2 therefore may have growth inhibitory activity on tumor cells. Additionally MMP-9, through its extracellular remodeling activities, may facilitate the immigration of tumor cells into the pulmonary environment (Coussens and Werb, 1996; Werb et al., 1999). Prior studies demonstrating that CHI3L1 promotes both macrophage recruitment and angiogenesis in colorectal cancer (Kawada et al., 2012) lend support to the idea that CHI3L1 expressed by interstitial and alveolar lung macrophages in the mammary tumor-bearing mice may likewise promote the migration and growth of metastasizing tumor cells.

At metastatic sites, specific populations of myeloid cells, i.e., CD11b^+^Gr1^+^ cells, have been found to promote tumor cell extravasation, seeding and persistent growth (Qian et al., 2009, 2011; Yan et al., 2010). The effect of a primary tumor affecting a distant organ such as the lung was previously investigated by (Yan et al., 2010), and it was found that CD11b^+^Gr1^+^ cells are increased in number in the “pre-metastatic” lungs of mice with mammary tumors (Yan et al., 2010). We have previously shown that splenic CD11b^+^Gr1 cells express CHI3L1. Since Gr1 marker is a composite epitope between Ly6C and LyG antigens, we assessed the expression on CHI3L1 in CD11b^+^Ly6C^+^ vs. CD11b^+^Ly6G^+^ populations of cells from total lung and the lavage. We demonstrate that CD11b^+^Ly6C^+^ populations in the lungs of tumor bearers produce high levels of CHI3L1. In the study by Yang et al. (2004) CD11b^+^Gr1 cells in the pre-metastatic lung down-regulated IFN-γ, contributing to the immunosuppressive stage, and we have shown previously that CHI3L1 downregulates IFN-γ expression by splenic T cells (Libreros et al., 2012). Additionally, use of CHI3L1 knockout mice in an allergic pulmonary model was shown to increase IFN-γ in comparison to levels in wild type mice with normal CHI3L1 expression (Lee et al., 2009). It may be speculated that CHI3L1 in the pulmonary tissue may have similar effects on local IFN-γ expression, and thus contribute toward the establishment of metastases in our mammary tumor bearers. Chitin microparticle suppression of CHI3L1 could counteract this, and our prior findings of increased IFN-γ levels in chitin microparticle-treated tumor-bearers is consistent with this hypothesis, as these mice show decreased mammary tumor growth (Libreros et al., 2012). Decreased tumor burden is known to affect the success of tumor cell metastasis to peripheral organs. Binding of chitin to CHI3L1 may neutralize the adverse effects of CHI3L1 on tumor growth and metastasis both by decreasing angiogenesis and increasing IFN-γ expression. Thus, understanding how molecules like CHI3L1, expressed in the target organ at “pre-metastatic” stages, can promote the establishment of cancer cells at these target sites, may provide insights about how to disrupt these mechanisms therapeutically. Chitin microparticles may represent one possible method to neutralize the adverse effects of endogenous CHI3L1 on cancer cell growth, particularly in an inflammatory tissue environment.

### Conflict of interest statement

The authors declare that the research was conducted in the absence of any commercial or financial relationships that could be construed as a potential conflict of interest.
